# Spontaneous Rupture of the Urinary Bladder 10 Years after Curative Radiotherapy

**DOI:** 10.1100/tsw.2008.67

**Published:** 2008-04-20

**Authors:** Apostolos P. Labanaris, Vahudin Zugor, Reinhold Nützel, Reinhard Kühn

**Affiliations:** ^1^ Department of Urology, Martha Maria Medical Center, Nurnberg, Germany; ^2^ Department of Urology, University of Erlangen Medical Center, Erlangen, Germany

**Keywords:** spontaneous bladder rupture, diagnosis, treatment, incidence

## Abstract

It has been reported that the rate of clinically relevant side effects following curative radiotherapy for primary carcinoma is about 3% for urologic complications. Such complications include hematuria, fibrosis, and cystitis. An extremely rare, but dangerous, medical complication following curative radiotherapy that can also be noted is spontaneous bladder perforation. We present such a case of a 27-year-old patient with spontaneous bladder perforation, who was initially misdiagnosed because of its rarity as well as unspecific clinical and laboratory findings.

## INTRODUCTION

Spontaneous rupture of the urinary bladder is an uncommon, but dangerous, medical emergency that can be fatal if its diagnosis and treatment are delayed or missed. It is a rare clinical manifestation that is nearly always provoked by underlying bladder conditions that are inflammatory, malignant, or obstructive[1]. These conditions, which in most reported cases include infravesical obstruction, bladder tuberculosis, prolonged cystitis, bladder diverticulum, bladder carcinoma, but also pelvic irradiation, weaken the bladder wall and precipitate the perforation[2].

## CASE REPORT

A 27-year-old woman was admitted to an Emergency Medicine department with the chief complaint of generalized abdominal pain associated with nausea and vomiting. Her pain had initially started suddenly in the suprapubic region after urination. She reported normal bowel function and denied abdominal trauma. Before that time, she had no urological complaints, but stated that she urinates seven to nine times a day. Urinalysis demonstrated mild microhematuria without signs of infection. She had a history of a primary pelvic Ewing's sarcoma 10 years ago, treated with curative radiochemotherapy. No significant complications had been reported and no evidence of disease recurrence had been documented in routine follow-up since that time.

At the time of her admission, examination revealed a distressed, but hemodynamically stable, patient. Her abdomen was lightly distended due to ascites and intestinal gas. She complained of severe tenderness of the whole abdomen without significant muscular defense and rebound tenderness. Bowel sounds were absent. The bladder was catheterized and about 80 ml of urine was obtained. Ultrasound examination described an insignificant collection of intra-abdominal fluid and no abnormal lesions were identified elsewhere in the abdomen or pelvis. Abdominal X-ray films of the abdomen suggested no evidence of bowel obstruction or free air. CT scan showed mild ascites, but no other associated pathology. Laboratory findings exhibited a white blood count of 6.2 g/l, hemoglobin 13.6 g/l, serum creatinine elevation at a level of 4.3 mg/dl, and GFR was 17.8. Preoperative diagnosis was that of peritonitis and acute renal failure.

After 7 days of hospitalization and although the patient still catheterized with 400–500 ml of urine being obtained every 24 h, the patient exhibited progressive ascites and so a punction was performed with about 3 l of translucent yellow fluid being retrieved from the peritoneal cavity. Laboratory examination of the fluid led to the diagnosis of urinary ascites. After the ascites punction was performed, the patient showed great signs of improvement and 7 days later, she was discharged from the hospital with all clinical and laboratory findings returning to normal. Two weeks later, the patient was admitted to our department once more with generalized abdominal pain accompanied this time by urinary retention. Ultrasound examination, though, exhibited an empty urinary bladder. Immediately, cystoscopy accompanied by cystography was performed. Cystoscopic examination revealed an edematous mucosa with ulcer and trabeculation with a 1.2 × 1.3 perforation on the bladder dome within an attenuated area of 5 cm in diameter. The bladder capacity of the patient was less than 150 cc. Cystogram showed a massive intraperitoneal extravasation of contrast media (Fig. 1). The diagnosis made was spontaneous bladder rupture. The patient was, of course, prepared and surgery took place the next day. At laparotomy, there was no macroscopic evidence of intra-abdominal malignancy. The cystoscopic and cystographic results were verified. The bladder wall around the perforation was excised to include the area of attenuation and the bladder was augmented with small intestine. The bladder augmentation was decided because of the low bladder capacity as well as the age of the patient. Histologically, acute and chronic cystitis with focal abscess formation in the muscle layer was seen. Her postoperative recovery was uncomplicated.

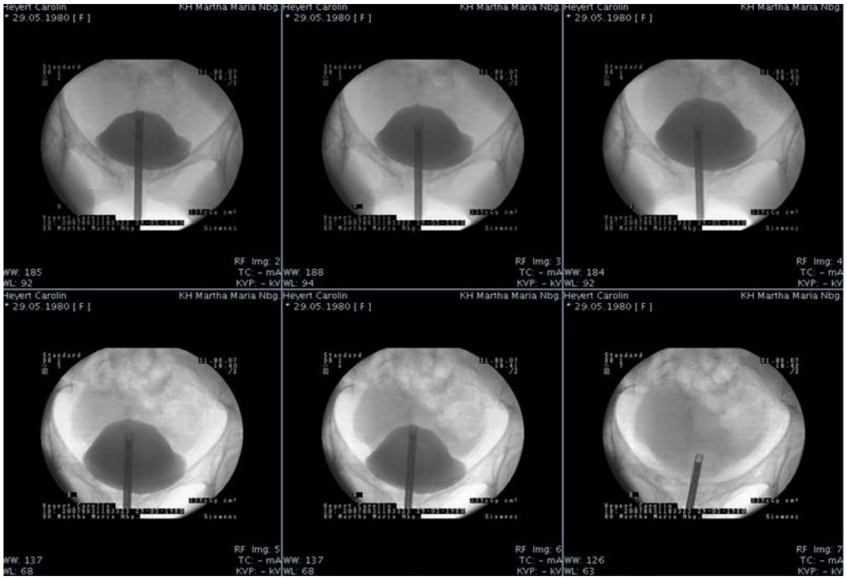

**FIGURE 1.** Cystogram showing a gradual massive intraperitoneal extravasation of contrast media.

## DISCUSSION

The application of high-dose radiotherapy to various pelvic types of cancer is possible, but adjacent organs, such as the bladder, ureter, and rectum, are subject to treatment side effects of varying severity. It has been reported that the rate of clinically relevant side effects following curative radiotherapy for primary carcinoma is about 10% overall and approximately 3% for urologic complications, and are related to the dose and fractionation of the delivered dose and the volume irradiated. Such complications include hematuria, fibrosis, cystitis, fistula formation, and contractions[3].

Radiation therapy induces a wide spectrum of pathological changes in the bladder. Abnormalities (including inflammatory infiltrates, fibrosis, cellular atypia, and necrosis) may be found in all layers of the bladder wall, as well as in the vasculature. These alterations may contribute to structural weakness that predisposes the irradiated bladder to spontaneous perforation[4].

In adults, the timing of these perforations has been reported as remotely as 15 years following treatment, and repeat episodes are known to occur and should be considered in the acute setting[5].

Although the first case of spontaneous bladder rupture associated with pelvic radiation in English literature was reported in 1966 by Altman and Horsburgh[6], its exact incidence is not well known. Fujikawa et al.[2] reported 2.0% cases of spontaneous bladder rupture in 271 Japanese patients with radiation-induced complications, but concluded that the sensitivity to radiotherapy may differ between Japanese and Americans/Europeans, or the technique of radiotherapy used may differ between areas, making this complication extremely rare in the U.S. and Europe. The reason for this difference is not clear.

The most frequent location for intraperitoneal perforation is in the dome or in the posterior wall of the bladder. This is elucidated by the fact that it is usually the weakest point of the bladder, or the dome, that lacerates when there is an increase of intravesical pressure and that the upper part of the bladder is intraperitoneal when it is distended[7].

Spontaneous intraperitoneal rupture occurs more frequently than spontaneous extraperitoneal rupture. The classical history of intraperitoneal bladder rupture is sudden onset of lower abdominal pain with impaired micturition. Physical examination may reveal signs of peritonitis, although the initial findings may be minimal[2]. To obtain the diagnosis of bladder rupture, cystography is the most sensitive and accurate technique[8]. As for other diagnostic methods, the usefulness of CT images using contrast media or ultrasonography has been reported, but they are not as sensitive as cystography. However, achieving an accurate diagnosis has still proved to be difficult. Mokoena and Naidu[9] reported that there was a mean delay of 5.4 days (range: 2 h to 36 days) between an identifiable associated incident or the first medical attendance and diagnosis.

As do the radiological diagnostic methods, so can laboratory findings point us to the right direction in spontaneous bladder rupture detection. There is a significant reabsorption of urea and creatinine through the peritoneum, causing a significant elevation in blood urea and creatinine levels[10]. In addition, the serum level of potassium increases, and sodium and chloride concentrations decrease. Most of these laboratory data are similar to those of acute renal failure, making it hard to distinguish it from intraperitoneal rupture of the urinary bladder[11].

Management of spontaneous bladder rupture following pelvic radiation therapy must be individualized of course, but should also be based on six principles as described by Addar et al.[1]: (1) the defect must be initially identified and confirmed, (2) the peritoneal cavity should be thoroughly inspected and lavaged, (3) the defect should be widely excised, (4) reconstitution of the intact bladder should be performed with tissue supplied by an intact blood supply, (5) adequate healing should be supported by prolonged bladder drainage and prophylactic antibiotics, and (6) primary or recurrent malignant disease should be excluded.

The mortality rate associated with a delay in diagnosis of 24 h or more is as high as 25% for spontaneous bladder rupture. However, the mortality rate may be minimized by appropriate preoperative diagnosis and prompt surgical correction[12].

In conclusion, the most important key to early and accurate diagnosis of bladder rupture is for physicians to suspect this disorder in patients with a history of receiving pelvic radiation therapy, with peritonitis or/and features of acute renal failure. The presence of urine in the urinary bladder by catheterization does not exclude this rare modality.
